# A174 PERCEPTIONS OF CANNABIS USE IN WOMEN WITH INFLAMMATORY BOWEL DISEASE OF REPRODUCTIVE AGE: A CROSS-SECTIONAL STUDY

**DOI:** 10.1093/jcag/gwab049.173

**Published:** 2022-02-21

**Authors:** P Tandon, K O’Connor, H Steinhart, A Deshpande, C Maxwell, V Huang

**Affiliations:** University of Toronto, Woodbridge, ON, Canada

## Abstract

**Background:**

Cannabis use in inflammatory bowel disease (IBD) may lead to improvement in pain and general health perception. However, its use during pregnancy may result in adverse outcomes such as preterm birth and altered fetal brain development. It remains unknown how women with IBD perceive Cannabis use during pregnancy and whether they discuss its use with their health-care providers.

**Aims:**

To determine practices in, and perceptions of, cannabis use during pregnancy in women with IBD of reproductive age.

**Methods:**

Women with IBD (age 18–45) were recruited at Mount Sinai Hospital and via social media platforms. Participants anonymously completed surveys on baseline demographics and IBD characteristics. They also completed a Cannabis questionnaire which asked about current use, perceived risks during pregnancy, and discussions with health-care providers. Categorical variables were reported as frequencies and compared using the chi-square test. Continuous variables were reported as medians and compared using the Mann-Whitney U test.

**Results:**

Sixty-four women were included, 26 (40.6%) with ulcerative colitis, 37 (57.8%) with Crohn’s disease, and 1 (1.6%) with indeterminate colitis. Nineteen (29.7%) were preconception, 40 (62.5%) were pregnant, and 5 (7.8%) were post-partum. Eleven (18.0%) patients reported current Cannabis use, 4 (6.3%) during pregnancy. Cannabis users were more likely to have discussed its use with a health-care provider compared to non-users (45.5% vs. 5.7%, p<0.001) and had longer IBD duration (12.00 vs. 9.00 years, p=0.05). Twenty-five (42.4%) were unsure of the risks of Cannabis use in pregnancy, of which only two had discussed this with a health-care provider. Reasons for fear of Cannabis use included risk of fetal oxygen restriction (n=13, 20.3%), impact on brain development (n=29, 46.0%), and risk of fetal respiratory issues (n=18, 28.6%). Only eight (12.5%) patients reported having a conversation about Cannabis use during pregnancy with their health-care provider; all of whom felt its consumption was unsafe during pregnancy.

**Conclusions:**

Many women with IBD report being unsure of risks of Cannabis use during pregnancy. With the legalization of Cannabis in Canada, it is imperative patients and health-care providers discuss the risks and benefits of its use, particularly during vulnerable times such as pregnancy.

**Funding Agencies:**

None

